# Surveying Cory Shearwater colonies with camera traps and identifying potential invasive nest predators

**DOI:** 10.3897/BDJ.11.e103270

**Published:** 2023-04-03

**Authors:** Lucas Lamelas-Lopez, Paulo A. V. Borges

**Affiliations:** 1 Centre for Ecology, Evolution and Environmental Changes (cE3c)/Azorean Biodiversity Group, CHANGE – Global Change and Sustainability Institute, Faculty of Agricultural Sciences and Environment, University of the Azores, Rua Capitão João d´Ávila, Pico da Urze, 9700-042, Angra do Heroísmo, Azores, Portugal Centre for Ecology, Evolution and Environmental Changes (cE3c)/Azorean Biodiversity Group, CHANGE – Global Change and Sustainability Institute, Faculty of Agricultural Sciences and Environment, University of the Azores, Rua Capitão João d´Ávila, Pico da Urze, 9700-042 Angra do Heroísmo, Azores Portugal; 2 IUCN SSC Mid-Atlantic Islands Invertebrates Specialist Group, Angra do Heroísmo, Azores, Portugal IUCN SSC Mid-Atlantic Islands Invertebrates Specialist Group Angra do Heroísmo, Azores Portugal

**Keywords:** biodiversity, biological invasions, camera-traps, invasive predators, inventory, Oceanic Islands, seabirds.

## Abstract

**Background:**

The Azores holds the largest population of Cory's shearwater *Calonectrisborealis* (Cory, 1881) (Aves, Procellariiformes, Procellariidae) in the world. One of the major threats of this species in the Azores is the predation by invasive mammals, which were introduced during European colonisation of the islands.

The present study provides a dataset from a camera-trapping survey performed in colonies of Cory’s shearwater. The sampling was conducted between 7 April and 23 October 2019, covering the entire breeding season, in three colonies of the Terceira Island (Azores). A total of 32 nests were sampled using motion-triggered cameras. The aims of this study are to provide information about the ecological patterns of the Cory shearwater and to identify potential nest predators.

**New information:**

Our results include a total of 6972 records of 15 species (nine species of birds, five of mammals and one reptile), of which 5414 records are of Cory’s shearwater, 478 of potential mammal predators and 1080 of another vertebrate species. Information about the biology of the species is also provided, as species circadian behaviour and habitat description.

## Introduction

Biological invasions, climate change and habitat fragmentation, degradation and destruction are the main drivers of biodiversity loss worldwide (e.g. Vitousek et al. (1997), Bellard et al. (2014), Doherty et al. (2016)). These three biodiversity erosion drivers can act synergistically, but invasive species alone can affect dramatically the native species communities and ecosystems functioning (e.g. Capizzi (2020)). In comparison with mainland areas, island ecosystems are especially vulnerable to biological invasions (Blumstein and Daniel 2005, Bellard et al. 2014, Spatz et al. 2017). Mammal predators constitute an important threat to island native vertebrates, being responsible by the decline or extinction of hundreds of island species worldwide (Medina et al. 2014, Dawson et al. 2014, Doherty et al. 2016). Island terrestrial and marine birds have been particularly affected by the introduction of invasive mammals (Medina et al. 2014, Spatz et al. 2017).

The Azores Archipelago comprises nine main islands of volcanic origin and it is located in the North Atlantic Ocean. The islands are considered a high priority area for seabird conservation, harbouring important populations of many seabird species, as for example, the globally endangered Monteiro's storm-petrel *Hydrobatesmonteiroi* Bolton et al. 2008 (Bolton et al. 2008, BirdLife-International 2016) or the Cory’s shearwater *Calonectrisborealis* (Cory, 1881), for which the Azores population is one of the largest worldwide.

Studies about seabirds and terrestrial birds’ populations in the Archipelago showed that mammal predators are probably the main cause of breeding failure (Monteiro et al. 1996, Amaral et al. 2010, Hervías et al. 2013a, Hervías et al. 2013b, Lamelas-López et al. 2020, Lamelas-López et al. 2021) or extinction (Monteiro et al. 1996). Mammals were introduced in the Archipelago as a consequence of the Portuguese arrival and settlement in the 15^th^ century. Currently, the mammal predators present in the Archipelago include rodents (house mouse *Musmusculus* Linnaeus, 1758, black rat *Rattusrattus* Linnaeus, 1758 and Norway rat *Rattusnorvegicus* Berkenhout, 1769) and carnivores (ferret *Mustelafuro* Linnaeus, 1758, weasel *Mustelanivalis* Linnaeus, 1766, feral cat *Felissilvestriscatus* Schreber, 1775 and feral dog *Canislupusfamiliaris* Linnaeus, 1758).

Identification of predators and the knowledge about their ecological patterns are crucial to the conservation of native terrestrial and marine birds of the Azores (Rader et al. 2007, Richardson et al. 2009). In this context, camera-trapping has been demonstrated to be an efficient tool to answer a variety of research questions in the fields of animal ecology, behavioural studies and conservation biology or for the inventory and monitoring of wildlife (Tobler et al. 2008, O'Connell et al. 2011, Rendall et al. 2014), particularly applied to identify invasive predators and to assess their impacts on native biodiversity (Oppel et al. 2014, Lamelas-López et al. 2020, Lamelas-López et al. 2021).

## General description

### Purpose

The main objectives of this study are to provide a dataset of species present in three Cory Shearwater colonies of Terceira Island, obtained from camera-trap records; and to obtain information about the biology of the Cory Shearwater, through the description of habitat and circadian behaviour and to identify potential introduced mammal predators.

## Project description

### Title

Surveying seabird colonies with camera traps: The impacts of invasive predators on Cory Shearwater

### Personnel

Lucas Lamelas-López, Paulo A.V. Borges

### Study area description

The study was conducted in three of Cory’s shearwater colonies, on Terceira Island (total area: 400.2 km²; maximum elevation: 1021 m a.s.l; -38°40'N, 27°10'W), which belongs to the Azores Archipelago (North Atlantic). Chanoca colony is located on the southern coast of the Island (maximum elevation: 21 m a.s.l; 38°39'36.0288"N, 27°17'42.1872"W) and it is formed by cliffs and rocky bays, slightly covered by some herbaceous plants, such as sour fig *Carpobrotusedulis* (L.) N.E. Br. Raminho colony (maximum elevation: 90 m a.s.l; 38°46'50.5668"N, 27°21'23.6736"W) is located in the north-west and it is characterised by cliffs dominated by native forests, mainly composed by *Ericaazorica* Hochst. ex Seub, and *Morellafaya* (Aiton) Wilbur. Finally, the Agualva colony (maximum elevation: 34 m a.s.l; 38°47'40.6068"N, 27°11'28.3452"W) is located in the north of the Island and it consists of a rocky area scarcely covered by patches of native vegetation (*E.azorica*).

### Design description

Motion-triggered infrared cameras (Bushnell Trophy HD, Moultrie 880i and 990i) were installed in the colonies at the beginning of the breeding season (e.g. Lamelas-López et al. (2020)). We installed one camera per nest, which remained recording continuously until the end of the breeding season or until the nest was abandoned or depredated and then the camera was moved to another nest. The cameras were deployed at 50-100 cm of the nest entrances. Cameras were configured to take 8 MB-photos, with 30 seconds of delay between them (Lamelas-López et al. 2021). Date and time were automatically recorded for each event. The nests were monitored each 10 days, in order to assess the nest condition and to replace the SD cards and the batteries of the cameras, if necessary. The study was conducted from 7 April and 23 October 2019. The obtained photos were posteriorly analysed and identified by L.L.L.

### Funding

Fieldwork: Fundação para a Ciência e Tecnologia - FCT (SFRH/BD/115022/2016)

Database management: FCT-UIDB/00329/2020-2024 (Thematic Line 1 – integrated ecological assessment of environmental change on biodiversity) and also FCT-UIDP/00329/2020-2023.

## Sampling methods

### Study extent

The study was conducted in three of Cory’s shearwater colonies, on Terceira Island (total area: 400.2 km²; maximum elevation: 1021 m a.s.l; -38°40'N, 27°10'W). Chanoca colony is located on the southern coast of the Island (maximum elevation: 21 m a.s.l; 38°39'36.0288"N, 27°17'42.1872"W) and it is formed by cliffs and rocky bays, slightly covered by some herbaceous plants such as sour fig *Carpobrotusedulis* (L.) N.E. Br. Raminho colony (maximum elevation: 90 m a.s.l; 38°46'50.5668"N, 27°21'23.6736"W) is located in the north-west and it is characterised by cliffs dominated by native forests, mainly composed by *Ericaazorica* Hochst. ex Seub and *Morellafaya* (Aiton) Wilbur. Finally, the Agualva colony (maximum elevation: 34 m a.s.l; 38°47'40.6068"N, 27°11'28.3452"W) is located in the north of the Island and it consists of a rocky area scarcely covered by patches of native vegetation (*E.azorica*).

### Sampling description

Motion-triggered infrared cameras (Bushnell Trophy HD, Moultrie 880i and 990i) were installed in the colonies at the beginning of the breeding season (e.g. Lamelas-López et al. (2020)). We installed one camera per nest, which remained recording continuously until the end of the breeding season or until the nest was abandoned or depredated and then the camera was moved to another nest. The cameras were deployed at 50-100 cm of the nest entrances. Cameras were configured to take 8 MB-photos, with 30 seconds of delay between them (Lamelas-López and Salgado 2020, Lamelas-López et al. 2021). Date and time were automatically recorded for each event. The nests were monitored each 10 days, in order to assess the nest condition and to replace the SD cards and the batteries of the cameras, if necessary. The study was conducted from 7 April and 23 October 2019. The obtained photos were posteriorly analysed and identified by L.L.L.

### Quality control

All the photos were carefully verified by the authors.

### Step description

Between 7 April and 23 October 2019, a total of 32 camera-traps were installed in three of Cory’s shearwater colonies on Terceira Island, covering the entire breeding period. We searched occupied nests and installed one camera per nest, which remained recording continuously until the end of the breeding season or until the nest was abandoned or depredated and then the camera was moved to another nest. Cameras were deployed at 50-100 cm of the nest entrance and were programmed to take photos, which recorded date and time of the event. Nests were monitored each 10 days, in order to assess the nest condition and to replace the SD cards and batteries of the cameras. The obtained photos were posteriorly analysed and identified by L.L.L.

The data have been published as a Darwin Core Archive (DwC-A), which is a standardised format for sharing biodiversity data as a set of one or more data tables. We provided an event data table, which contains 2976 records; and an occurrence data table, with 6972 records.

## Geographic coverage

### Description

Terceira Island, Azores, Portugal.

### Coordinates

 and 3843'17"N Latitude Latitude; 27°13'14"W Longitude and Longitude.

## Taxonomic coverage

### Description

The following Classes and Orders are covered: Aves: Procellariiformes, Columbiformes, Passeriformes; Mammalia: Carnivora, Lagomorpha, Rodentia; Reptilia: Squamata.

### Taxa included

**Table:** 

Rank	Scientific Name	Common Name
class	Aves	Birds
class	Mammalia	Mammals
class	Reptilia	Reptiles
order	Procellariiformes	Petrels
order	Columbiformes	Doves
order	Passeriformes	Passerines
order	Carnivora	Carnivores
order	Lagomorpha	Rabbits
order	Rodentia	Rodents

## Temporal coverage

**Data range:** 2019-4-07 – 2019-10-23.

## Usage licence

### Usage licence

Creative Commons Public Domain Waiver (CC-Zero)

## Data resources

### Data package title

Camera-traps_Seabirds_2019

### Resource link

http://ipt.gbif.pt/ipt/resource?r=camera-trap_seabirds_2023

### Alternative identifiers

https://www.gbif.org/dataset/7fa446fd-caf6-43a4-83f6-b2cbb06c51c7

### Number of data sets

2

### Data set 1.

#### Data set name

Event Table

#### Data format

Darwin Core Archive

#### Character set

UTF-8

#### Download URL

http://ipt.gbif.pt/ipt/resource?r=camera-trap_seabirds_2023

#### Data format version

version 1.2

#### Description

The dataset is available on the Global Biodiversity Information Facility platform, GBIF (Lamelas-López and Borges 2023). The following data table includes records at species level. The dataset submitted to GBIF is structured as a sample event dataset, with two tables: event and occurrence tables. The data in this sampling event resource have been published as a Darwin Core Archive (DwCA), which is a standardised format for sharing biodiversity data as a set of one or more data tables. The event table contains 2976 records. This IPT (Integrated Publishing Toolkit) archives the data and, thus, serves as the data repository. The data and resource metadata are available for download from Lamelas-López and Borges (2023).

**Data set 1.** 

Column label	Column description
id	Unique identification code for sampling event data.
eventID	Identifier of the events, unique for the dataset.
samplingProtocol	The sampling method used to obtain the records.
sampleSizeValue	The number of days that the cameras remain active in each sampling.
sampleSizeUnit	The unit of the sample size value.
eventDate	Date or date range the record was collected.
year	Year of the event.
month	Month of the event.
day	Day of the event.
habitat	The habitat type in which the event occurred.
fieldNotes	Notes about the use or non-use of bait in the sampling sites.
locationID	Identifier of the location.
islandGroup	Name of archipelago.
island	Name of the island.
country	Country of the sampling site.
countryCode	ISO code of the country of the sampling site.
stateProvince	Name of the region of the sampling site.
municipality	Municipality of the sampling site.
locality	Name of the locality.
decimalLatitude	The geographic latitude, in decimal degrees.
decimalLongitude	The geographic longitude, in decimal degrees.
geodeticDatum	The ellipsoid, geodetic datum or spatial reference system (SRS) upon which the geographic coordinates given in decimalLatitude and decimalLongitude are based.
coordinateUncertaintyInMeters	Uncertainty of the coordinates, in metres.
coordinatePrecision	Precision of the coordinates.
georeferenceSources	A list (concatenated and separated) of maps, gazetteers or other resources used to georeference the Location, described specifically enough to allow anyone in the future to use the same resources.

### Data set 2.

#### Data set name

Occurrence Table

#### Data format

Darwin Core Archive

#### Character set

UTF-8

#### Download URL

http://ipt.gbif.pt/ipt/resource?r=camera-trap_seabirds_2023

#### Data format version

version 1.2

#### Description

The dataset is available on the Global Biodiversity Information Facility platform, GBIF (Lamelas-López and Borges 2023). The following data table includes records at species level. The dataset submitted to GBIF is structured as a sample event dataset, with two tables: event and occurrence tables. The data in this sampling event resource have been published as a Darwin Core Archive (DwCA), which is a standardised format for sharing biodiversity data as a set of one or more data tables. The occurrence table contains 6972 records. This IPT (Integrated Publishing Toolkit) archives the data and, thus, serves as the data repository. The data and resource metadata are available for download from Lamelas-López and Borges (2023).

**Data set 2.** 

Column label	Column description
id	Unique identification code for species abundance data.
institutionID	The identity of the institution publishing the data.
institutionCode	The code of the institution publishing the data.
datasetName	Name of the dataset.
basisOfRecord	The nature of the data record.
occurrenceID	Identifier of the record, coded as a global unique identifier.
organismQuantity	A number or enumeration value for the quantity of organisms.
organismQuantityType	The type of quantification system used for the quantity of organisms.
behaviour	Information about the circadian activity of the individuals.
establishmentMeans	The process of establishment of the species in the location, using a controlled vocabulary: 'native', 'introduced', 'endemic', 'Macaronesian native'.
occurrenceStatus	Information about the presence/absence of a taxon at a camera location.
eventID	Identifier of the events, unique for the dataset.
identifiedBy	Name of the researcher who performed the identification of the photos.
dateIdentified	Year of the identification of the photos content.
identificationRemarks	Additional information about species identity, according to species code on the Azorean Biodiversity Portal (https://azoresbioportal.uac.pt/).
scientificName	Complete scientific name including author and year.
kingdom	Kingdom name.
phylum	Phylum name.
class	Class name.
order	Order name.
family	Family name.
genus	Genus name.
specificEpithet	Specific epithet.
infraspecificEpithet	Infraspecific epithet.
taxonRank	Lowest taxonomic rank of the record.
scientificNameAuthorship	Name of the author of the lowest taxon rank included in the record.

## Additional information

A total of 6972 records of vertebrates were obtained, belonging to three classes, seven orders, 11 families and 15 species (Table 1). Nine species of birds were recorded, of which four are considered Azorean endemic subspecies (*Columbapalumbusazorica* Hartert, 1905; *Fringillacoelebsmoreletti* Pucheran, 1859; *Sylviaatricapillagularis* Alexander, 1898; and *Turdusmerulaazorensis* Hartert, 1905), two native non-endemic (*Calonectrisborealis* (Cory, 1881); and *Erithacusrubecularubecula* (Linnaeus, 1758), one Macaronesian endemic (*Serinuscanaria* (Linnaeus, 1758)) and two introduced (*Columbaliviadomestica* Gmelin, 1758; and *Passerdomesticusdomesticus* Linnaeus, 1758) (Borges et al. 2010). Five species of mammals were detected, namely *Feliscatus* Linnaeus, 1758; *Musmusculus* Linnaeus, 1758; *Mustelanivalis* Linnaeus, 1766; *Oryctolaguscuniculus* (Linnaeus, 1758); and *Rattusrattus* (Linnaeus, 1758), which are all introduced species in the Azores. Finally, we recorded one single species of reptile, *Teiradugesii* (Milne-Edwards, 1829), which is also introduced coming from Madeira (native range).

Most of records (n = 5414) were of *C.borealis*, given that the cameras were deployed focusing on nest entrances. The most abundant bird species detected were *E.r.rubecula* (n = 245) and *T.merulaazorensis* (n = 432), which are native and endemic species, respectively. Introduced bird species showed low abundance (*C.liviadomestica* n = 13 records; *P.d.domesticus* n = 21 records). This is probably associated with the habitat types, given that native bird species are more frequent in native vegetation areas, as are the studied areas, while introduced bird species are commonly associated with more human-disturbed habitats.

**Figure 1.**
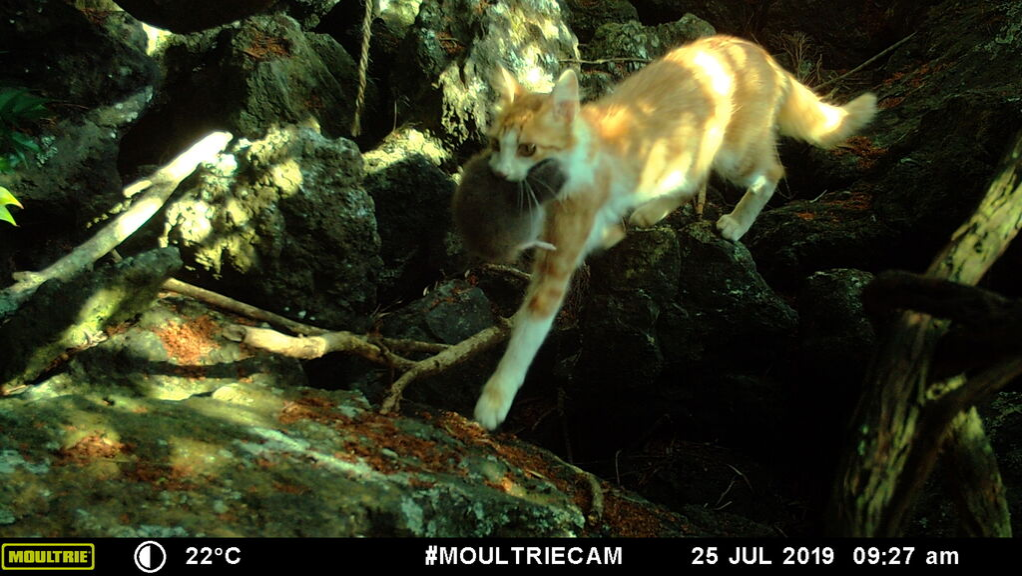
Record of *Feliscatus* predating on a chick of *Calonectrisborealis* in Chanoca colony.

Most abundant mammal species were rodents *R.rattus* (n = 294) and *M.musculus* (n = 110) and the domestic cat (n = 68). These species were detected in all *C.borealis* colonies and they are known predators of terrestrial birds and seabirds in many islands worldwide (Bolton et al. 2008, Medina et al. 2014; Spatz et al. 2017) and particularly in the Azores islands (Monteiro et al. 1996, Hervías et al. 2013a, Hervías et al. 2013b, Lamelas-López et al. 2020, Lamelas-López et al. 2021; Fig. 1). *M.nivalis* has also been reported has a potential predator of native birds in the Archipelago, but our data suggest that the impact will be probably low (we only recorded four events in one colony).

*Teiradugesii* was detected in the colonies (n = 298), mainly in the Chanoca colony, which is dominated by rocky areas.

Additionally, in the dataset, we also provided information about the behaviour of the species, particularly of the circadian activity of the species. *Calonectrisborealis* demonstrated to be more active during the dawn and dusk (n = 1738 records) and night (n = 3235 records), in comparison with day (n = 441 records). In general, introduced mammal predators were also more frequently observed during these periods. For example, *R.rattus* was mainly detected during the night (n = 217) or crepuscule (n = 64) in comparison with the day (n = 13). However, the *F.catus* was detected during all of the day (crepuscule n = 26, night n = 21, day n = 21).

Identification of introduced predator species and information of their abundance, habitat preferences or behaviour are crucial for information to design effective management plans and conservation actions (Thompson 2007, Lamelas-López et al. 2020).

## Figures and Tables

**Table 1.** Abundance, colonisation status (CS) and IUCN categories (IUCN) of species recorded in the three Cory’s shearwater colonies of Terceira Island (Azores), in 2019, based on camera-trapping data. Abbreviations: endemic subspecies of Azores (end); endemic of Macaronesia (mac); introduced (int); native non-endemic (nat); LC Least Concern; AGU Agualva Colony, CHA Chanoca colony, RAM Raminho colony.

Class	Order	Species	CS	IUCN	AGU	CHA	RAM
Aves	Columbiformes	*Columbaliviadomestica* Gmelin, 1758	int	LC	0	13	0
Aves	Columbiformes	*Columbapalumbusazorica* Hartert, 1905	end	LC	2	2	13
Aves	Passeriformes	*Fringillacoelebsmoreletti* Pucheran, 1859	end	LC	1	3	7
Aves	Passeriformes	*Serinuscanaria* (Linnaeus, 1758)	mac	LC	1	0	1
Aves	Passeriformes	*Passerdomesticusdomesticus* Linnaeus, 1758	int	LC	1	16	4
Aves	Passeriformes	*Sylviaatricapillagularis* Alexander, 1898	end	LC	33	0	8
Aves	Passeriformes	*Erithacusrubecularubecula* (Linnaeus, 1758)	nat	LC	34	0	211
Aves	Passeriformes	*Turdusmerulaazorensis* Hartert, 1905	end	LC	103	7	322
Aves	Procellariiformes	*Calonectrisborealis* (Cory, 1881)	nat	LC	939	1801	2674
Mammalia	Carnivora	*Feliscatus* Linnaeus, 1758	int	LC	29	27	12
Mammalia	Carnivora	*Mustelanivalis* Linnaeus, 1766	int	LC	4	0	0
Mammalia	Lagomorpha	*Oryctolaguscuniculus* (Linnaeus, 1758)	int	LC	2	0	0
Mammalia	Rodentia	*Musmusculus* Linnaeus, 1758	int	LC	4	82	24
Mammalia	Rodentia	*Rattusrattus* (Linnaeus, 1758)	int	LC	45	29	220
Reptilia	Squamata	*Teiradugesii* (Milne-Edwards, 1829)	int	LC	15	283	0
